# The Effect of Ageing in Water Solution Containing Iron Sulfate on the Mechanical Properties of Epoxy Adhesives

**DOI:** 10.3390/polym12010218

**Published:** 2020-01-15

**Authors:** Anna Rudawska, Valentina Brunella

**Affiliations:** 1 Faculty of Mechanical Engineering, Lublin University of Technology, 20-618 Lublin, Poland; 2 Department of Chemistry of the University of Turin, Via P. Giuria 7, 10125 Torino, Italy; valentina.brunella@unito.it

**Keywords:** epoxy adhesive compounds, ageing, water solution, iron sulfate, mechanical properties

## Abstract

This study investigates the effect of operating factors such as seasoning in water solution containing iron (II) sulfate—FeSO_4_ (5 different water solution variants were tested) on the mechanical properties of an adhesive compound made of epoxy resin and amine curing agent, in a ratio of 100 g resin to 12 g curing agent. Strength tests of cured adhesive compound samples were performed on the Zwick/Roell Z150 testing machine in compliance with the EN ISO 604 standard. During the tests, compression modulus, compressive strength and compressive strain were measured. Obtained results served as a basis for analyzing the effect of a water environment containing iron sulfate on a given adhesive compound. It has been found that too high iron sulfate content in water has a negative effect on the mechanical properties of adhesive compound samples.

## 1. Introduction

Nowadays, adhesive-bonded joints are used in many industries such as automotive, building, aircraft and civil infrastructures [1,2,3,4,5]. It is essential that adhesive-bonded joints have a high amount of strength, in addition to showing good adhesion during joint-making. Thanks to the use of adhesives, bonded elements are resistant to failure, and the use of a suitable adhesive bonding technique ensures the achievement of the highest possible strength of the bonded elements. Adhesives enable us to reduce structure weight to a significant extent. As a result, it is possible to use lightweight construction materials, which have had widespread use in the aircraft industry for years. Another advantage of adhesives is their vibration damping capability, a feature that is extremely desired in industries such as automotive or aircraft. What is more, it is possible to bond two completely different adherends, and no electrochemical corrosion will occur because the adhesive acts as an insulator for bonded elements [2,3,4,6].

Currently, there are numerous studies investigating the possibility of increasing adhesive strength properties and selecting the best modifiers so that adhesive compounds have the highest possible strength and properties under given operating conditions. All these activities help to improve the properties of adhesives and adherends alike. Incorrectly made adhesive-bonded joints have lower strength. Adhesives are mainly described with parameters such as viscosity, curing time, open drying time and chemical basis [7,8]. Moreover, adhesives must be resistant to external (operating) factors, e.g., the impact of the environment in which they are used [9,10,11,12,13,14,15,16]. Such environment can either improve or worsen adhesive properties. One of the operating factors is the water environment [17,18,19,20]. 

Some researchers have presented the results of the impact of humid ageing on the strength of adhesive and adhesive joints [16,21,22,23,24,25,26]. Leger et al. [2] investigated the ageing of a single lap joint and one-part rubber toughened epoxy/amine adhesive in water at 70 °C. The impact of the temperature and water on the classic tensile data were evaluated during the tensile tests of bulk adhesive dog-bone specimens. The time of ageing the adhesive specimens was different. One kind of specimen was immersed in water at 70 °C during 30 min, 1 h, 3 h, 6 h, 1 day, 5 days and 15 days. In other work, Leger et al. [27] performed the tests on industrial rubber toughened epoxy adhesives to characterize its diffusion behavior in water at various temperatures. Sugiman et al. [15] considered the effect of moisture on the fatigue response of some types of adhesively bonded joints. The joints were aged in deionized water at 50 °C for up to 2 years exposure. The test results show that the fatigue life degraded with increasing moisture content and tended to level off when approaching saturation. Liechti et al. [28] presented that, in the air and in salt water, raising the temperature reduced the threshold energy release rate, and this decrease was more in salt water than in air. The results of the effects of environmental factors on the long-term performance of adhesive joints depicted in the works prepared by Pethrick [6], Heshmati et al. [16] and Karbhari et al. [5] underlined that moisture (in the form of humidity), liquid water or de-icing salt solutions are the most damaging substances. Bellini et al. [14] investigated the combined effect of hydrothermal ageing and the temperature test on the lap shear strength of single lap joints that are realized in CFRP (carbon fiber reinforced polymer). The results showed a higher influence of the ageing on paste adhesive compared to film adhesive. However, the ageing, combined with the operating temperature, played a fundamental role on the shear strength of the bonded joints. In work presented by Viana et al. [29], double cantilever beam (DCB) specimens using two different epoxy adhesives in the automotive industry were subjected to two different ageing environments: saturated solution of NaCl at 32.5 °C and distilled water at 32.5 °C. The results showed that the diffusion of water into the studied adhesive joints was faster than diffusion through the bulk adhesive alone. Ocaña et al. [4] studied the degradation process of adhesive joints of composite based on aluminum with two different adhesives (an epoxy and a polyurethane). During these tests a prolonged environmental exposure occurs and, moreover, ageing is caused by high temperature and immersion in the engine oil. Test results indicated that epoxy adhesive has better mechanical behavior than polyurethane when the stays have prolonged weathering. De Neve and Shanahan [17] investigated the ageing of a commercial epoxy adhesive based on DGEBA cured with dicyandiamide (DDA) and containing fillers in water vapor (ca. 100% RH) at various elevated temperatures. The results indicated that long-term exposure of the epoxy adhesive to water leads to both reversible (physical) and irreversible (chemical) degradation of the material. Yang et al. [30] presented the results of a durability program designed to study the effects of ageing and environment on the durability of a typical adhesive used in external bonding. The results showed that exposure to moisture causes plasticization and a decrease in performance characteristics. Lettieri and Frigione [24] investigated the effects of exposure to different humid environments in a commercial cold-cured epoxy adhesive. Samples of epoxy resin were exposed up to one month to a controlled humidity level (55% and 75% RH), kept in a saturated water vapor atmosphere (100% RH) or immersed in liquid water at a constant temperature (23 °C). Plasticization, reactivation of curing reactions and erasure of physical ageing were observed in the specimens subjected to the different humidity regimes and all affected both the thermal and the mechanical properties of the aged samples. Fernandes et al. [31] determined the fracture envelope of an adhesive as a function of the water content. The results showed that the toughness of the adhesive changed as a function of the ageing environment. For the salt water environment, the mechanical properties increased, while for the distilled water environment, degradation of the mechanical properties was observed. 

In water environments, iron compounds can usually be found in deep wells. Water from deep water wells flows around rocks collecting numerous minerals. The highest content of iron in water comes from the minerals of magmatic or sedimentary rocks. The presence of iron in water results from the fact that water has no contact with oxygen; the deeper the well is, the less oxygen in water and the higher the iron concentration. The presence of iron in water can also be a result of impurities that get into water, e.g., liquid wastes from metallurgical plants and mines, as well as impurities resulting from the corrosion of iron water tanks. Iron compounds in a water environment come in two forms: (i) bivalent iron (II) compounds (Fe^2+^) and (ii) rivalent iron (III) compounds (Fe^3+^) [32].

Water from deep water wells, deprived of oxygen, contains soluble bivalent compounds Fe^2+^, whereas oxygen-rich water contains soluble trivalent compounds Fe^3+^. The content of iron in a deep water well amounts up to several mg/L of water. This content depends on geological structures and the content of chemical compounds in rocks. Iron compounds rarely occur in surface waters, and their content merely amounts to mg/L. To de-iron bivalent water, a process aimed at obtaining water with trivalent iron compounds is carried out. A characteristic of trivalent iron compounds is a deposit that precipitates under the action of water. If the iron content is too high, this may cause sewage pipe clogging, yellow stains on objects in contact with water, not to mention the fact that it has a negative effect on the human body [32]. 

The objective of this study was to determine the effect of one of the operating factors, i.e., seasoning in water solution containing iron (II) sulfate—FeSO_4_ (using five water solution variants and a reference variant) on the mechanical properties of an epoxy adhesive compound.

## 2. Materials and Methods 

### 2.1. One-Factor Test Plan

**Figure 1:**
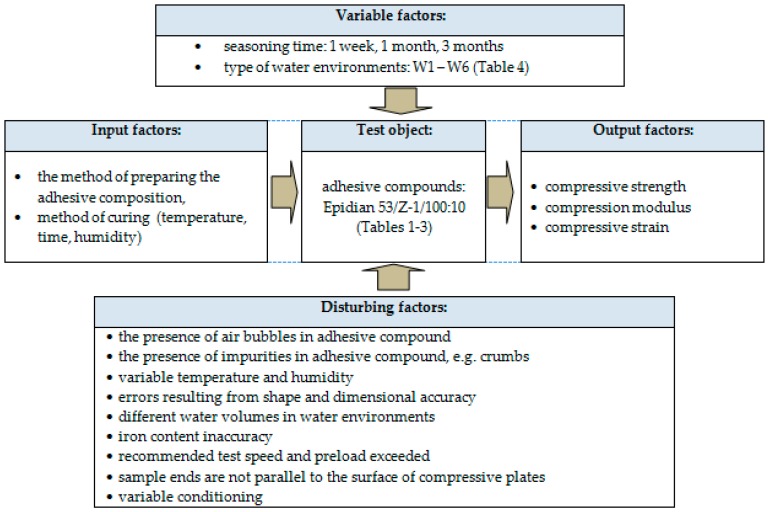
One-factor test plan.

Experiments were conducted in accordance with the one-factor test plan given below (Figure 1). The plan describes all of the factors that might affect the test results. Materials used in the experiments are described in subsequent sub-sections.

### 2.2. Epoxy Adhesive Compounds and Its Properties

Tests were performed on samples of an adhesive compound containing epoxy resin based on Bisphenol A (Epidian 53—trade name, manufactured by Organika-Sarzyna, Nowa Sarzyna, Poland) with a triethylenetetramine (TETA) curing agent (Z-1—trade name, manufactured by Organika-Sarzyna, Nowa Sarzyna, Poland) in a stoichiometric ratio of 100:10. On combining the two components, the entire adhesive compound progressed from a liquid state to a solid state. The following designation of adhesive compounds was adopted in the tests: Epidian 53/Z-1/100:10. The description of the epoxy resin and curing agent was presented in the [33].

The properties of Epidian 53 epoxy resin prior to curing are given in Table 1. The properties of Epidian 53 epoxy resin after curing are listed in Table 2.

Epidian 53 epoxy resin is used for adhesive bonding of glass, metal and ceramic elements as well as thermosetting plastics. Apart from being used as a basic component of adhesive compounds, it is also used for making casts and laminates in the optical or aircraft industry. This resin is characterized by low viscosity, relatively low reactivity, good adhesion with other materials, room temperature curing ability and very good electro-insulating properties. Epidian 53-based adhesives must be stored in tightly sealed containers, in a dry and airy room, away from direct sunlight. 

Epidian 53 epoxy resin is mixed with a suitable amount of the curing agents, and in this particular case, the triethylenetetramine (TECZA) curing agent (Z-1 trade name). It is one of the most popular amino curing agents used in compounds with low molecular weight epoxy resins. Properties of this amine curing agent are listed in Table 3. 

The curing process starts when the hardener is mixed together with the resin component. This moment also marks the gel time of a mixed resin system, i.e., the pot life of the curing resin system. It is vital that the epoxy resin/curing agent ratio be exact during mixing. A too high content of the curing produces strong reactions such as an exothermic reaction that—in extreme cases—may cause self-ignition of the compound. This leads to decreasing the strength properties of the polymer being cured. On the other hand, the low amine content leads to a lower resistance to temperature and chemical substances, as well as decreased dielectric properties of the compound. The curing process proceeds at approx. 25 °C. Pre-cure occurs after 3–4 h; after 2 days the samples show 80–90% of the total cure. 

### 2.3. Ageing Environments

For the purpose of the experiments, six water environments were prepared, including tap water containing iron (II) sulfate–FeSO_4,_ and mineral water “Zuber.” These water environments were denoted as specified below (Table 4). Table 5 gives selected physical and chemical parameters of tap water used in the experiments.

The water used in the experiments is bicarbonate-calcium-magnesium water; it is also hard water, as the content of calcium carbonates amounts to 381 mg/L (340–510 mgCaCO_3_/L—hard water). A characteristic of this type of water is its hardness resulting from the presence of dissolved minerals, primarily carbonates, chlorides, sulfates, magnesium, etc. The tap water was mixed with pure iron (II) sulfate (Biomus, Polish distributor, https://www.sklep.biomus.eu/pl), the physical and chemical properties of which are given below (Table 6) [36].

“Zuber” mineral water was selected for the experiments because it has the highest content of iron and minerals out of all mineral waters in Poland. This mineral water is drawn from four wells in Krynica Zdrój (a city in Poland), in the vicinity of Park Mountain (Góra Parkowa—polish name). The deepest water well is 935 m deep. This mineral water has healing properties; it has an anti-inflammatory effect and can be used in the treatment of metabolic disorders and digestive system problems. This mineral water must be stored at a temperature between 4 °C and 20 °C [37]. The total content of all dissolved minerals is 24,104.5 mg/dm^3^ (Table 7).

Following the preparation of water solutions, pH values of every tested water solution were measured using a universal indicator. The indicator was immersed in every water solution for about 5 s prior to reading the pH value [35]. Obtained results are listed in Table 8. 

Results (Table 8) demonstrate that after the addition of FeSO_4,_ the water pH changes from neutral to acidic. When increasing the content of iron (II) sulfate, the acidification of water increases too.

### 2.4. Shape and Dimensions of Samples

**Figure 2:**
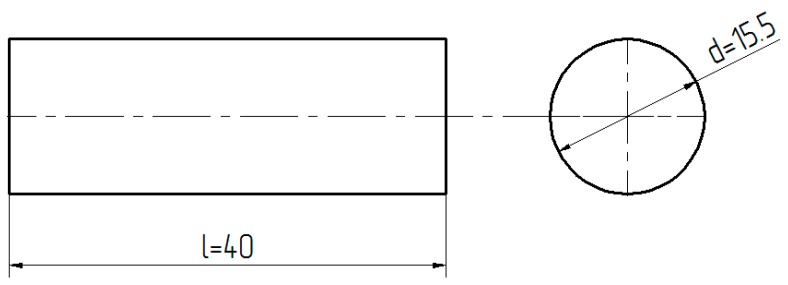
Shape and theoretical dimensions of adhesive samples.

**Figure 3:**
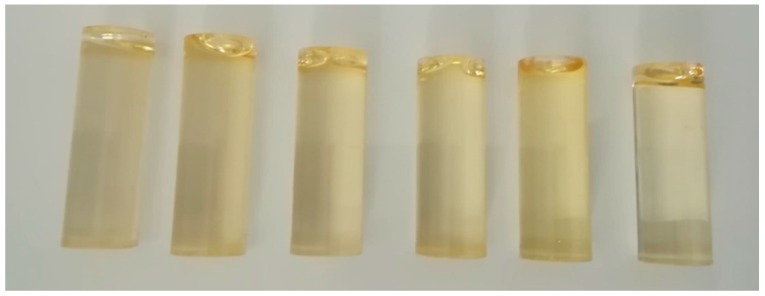
Cured adhesive compounds samples.

To ensure identical shape of adhesive samples, cylindrical molds with a capacity of 10 mL were used. This mold had a transparent barrel made of polypropylene. Figure 2 shows the shape and theoretical dimensions of the test samples, and Figure 3 shows examples of cured adhesive samples after removal from the mold.

Real dimensions of the samples depended on the accuracy of the measured adhesive amount in the mold. The amount of adhesive was approx. 8 mL, which—when converted into mm—amounted to a length 40 ± 1 mm and a diameter of about 15.5 ± 0.2 mm.

### 2.5. Technique and Conditions of Sample Preparation 

Samples were prepared in the following room conditions: temperature 20 ± 1 °C, and humidity 25 ± 1%. Sample preparation entailed measuring 100 g of Epidian 53 to 10 g of Z-1, with an accuracy of 0.1 g, using TP–2/1 scales (FAWAG S.A, Lublin, Poland). Next, both products were mechanically mixed with a horseshoe mixer in a polymer container on a specially designed stand for adhesive mixing. The rotational speed of the mixer was set equal to 460 rev/min, the mixing time was set to 2 min and the vacuum pump venting time was set equal to 2 min. 

Prior to putting the adhesive compounds in the molds, every mold was coated with an anti-adhesive agent in order to prevent adhesion of the adhesive compound to the mold. Known under the trade name of Polsilform (Polish Silicones, Nowa Sarzyna, Poland), this anti-adhesive agent creates an oily film with anti-adhesive properties on the mold surface. It is odorless and colorless. The agent was sprayed over the entire mold interior from a distance of 200 mm. 

Next, the mold was filled with approx. 8 mL of the adhesive compound. This operation required absolute precision to prevent the formation of air bubbles, which happens if the adhesive is batched either too slowly or too quickly. The adhesive compound samples were cold cured in the mold in a single operation at the ambient temperature of 20 ± 1 °C for 168 h. After curing, the samples were taken out from the molds and subjected to conditioning for 24 h at the ambient temperature of 20 ± 1 °C and humidity of 25 ± 1%. Next, the samples were put in six prepared water environments (Table 4), 15 samples per environment. The seasoning time of the six tested environments was as follows: (i) 1 week—5 samples, (ii) 1 month—5 samples and (iii) 3 months—5 samples.

**Figure 4:**
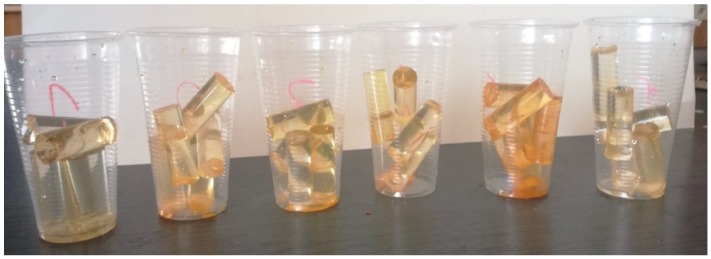
Adhesive compound samples after 3 months.

Below (Figure 4) shows the samples of adhesive compounds after 3 months of seasoning. 

**Figure 5:**
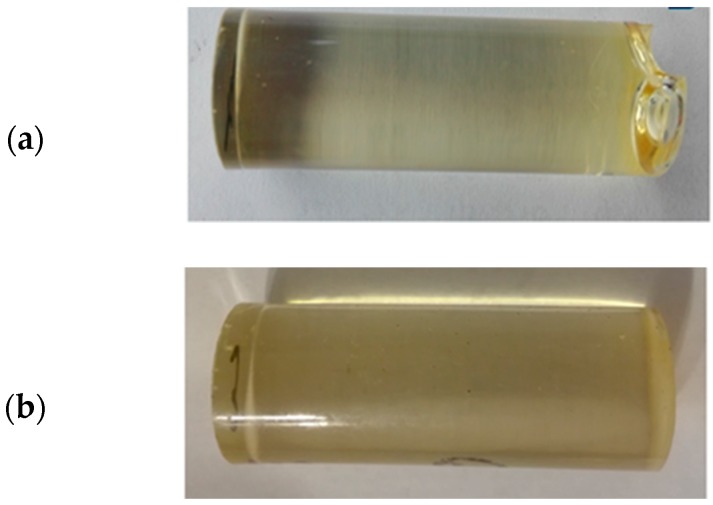
Adhesive sample: (**a**) before cutting, (**b**) after cutting.

Another stage of the study involved preparing the samples for strength tests. When taken out from the mold, some of the adhesive compound samples were found to have surface irregularities (Figure 5a), which results from the fact that air must have entered the mold. Adhesive compound samples were treated such that their end faces were parallel and lengths were comparable (Figure 5b). When doing this, it is essential that end faces be maintained parallel, because the samples were subjected to compression testing and any surface irregularity could distort results. To maintain the end faces parallel and make sample length their length, the samples were fixed in a vice and cut with an angular grinder. Post-cutting surface irregularities were removed by hand-grinding with P360-grade abrasive paper.

After treatment, the samples were weighed on a TP–2/1 scale (FAWAG S.A, Lublin, Poland) with an accuracy of up to 0.1. Next, the diameter and length of every sample were measured. The measurements were made using an electronic slide caliper with a measuring range of 0–150 mm and an accuracy of 0–100 mm ± 0.02 mm and 100–150 mm ± 0.03 mm.

### 2.6. Test Stand and Strength Testing 

Strength tests were performed on the Zwick/Roell Z150 machine (Zwick/Roell, Ulm, Germany) in compliance with the ISO 604 standard. They consisted in measuring the compressive strength of samples of the adhesive compound containing the Epidian 53 epoxy resin and the amine curing agent (Z-1) seasoned in different water environments containing iron (Table 4). The adhesive compound samples (Figure 3) were fixed in a stationary grip. It is essential that the test sample be located in the center of the stationary head. With the adhesive samples correctly positioned, compressive tests were performed at the following preset parameters: (i) force—30 N, (ii) compression modulus speed—2 mm/min and (iii) test speed—10 mm/min. The compressive tests were performed with the use of the testXpert testing software. 

## 3. Results and Discussion

**Figure 6:**
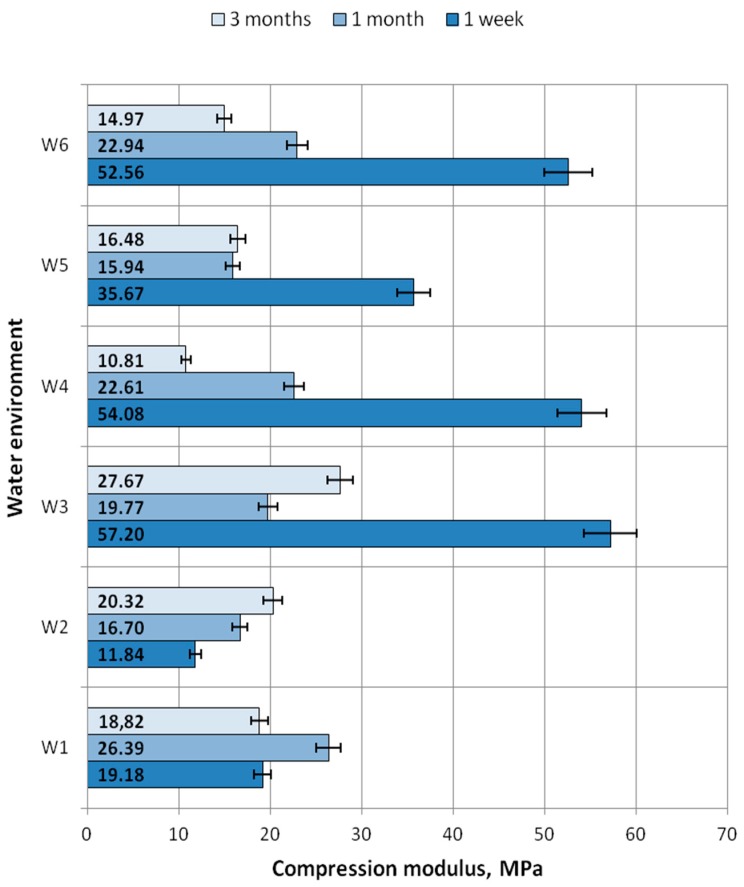
Compression modulus versus water environment and seasoning time.

**Figure 7:**
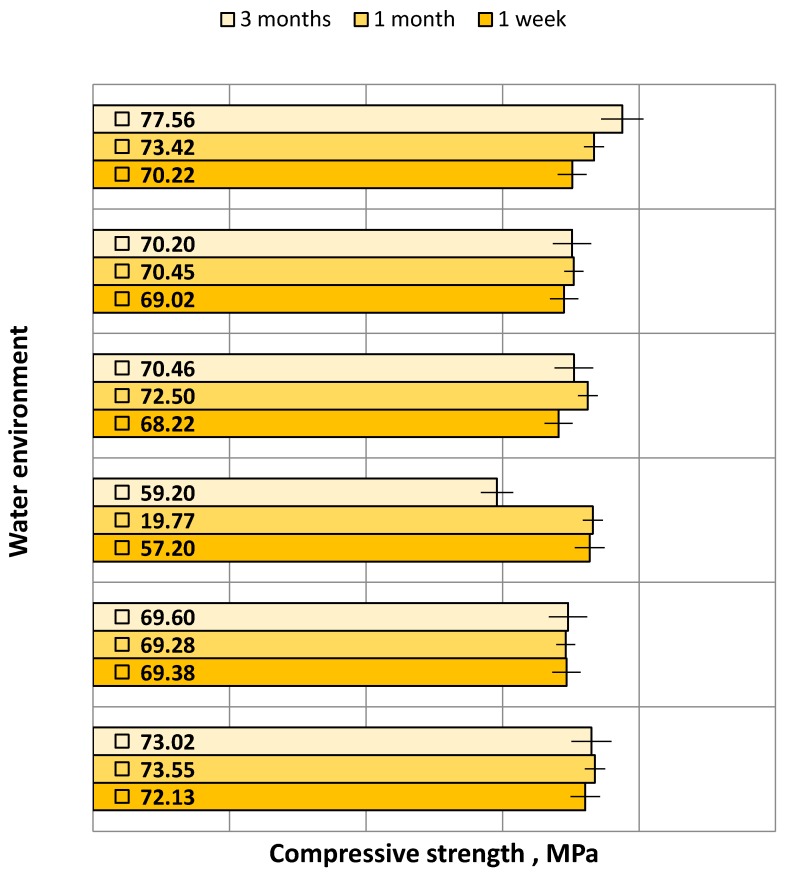
Compressive strength versus water environment and seasoning time.

**Figure 8:**
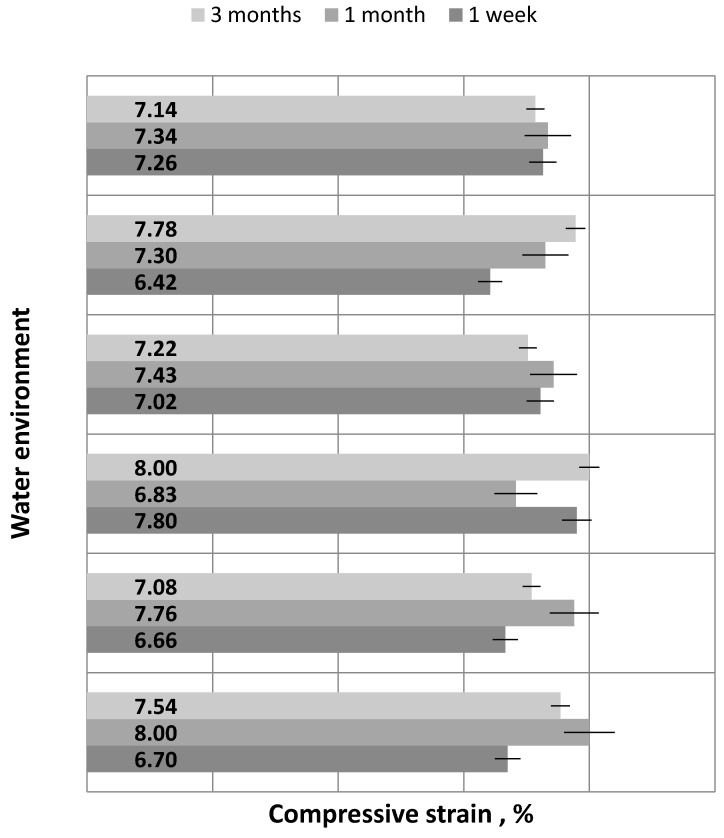
Compressive strain versus water environment and seasoning time.

Strength testing results were analyzed with respect to the type of water environment in which the samples of the Epidian 53/Z-1/100:10 adhesive compound were seasoned: 1 week, 1 month and 3 months, respectively. Obtained results were analyzed with respect to the following parameters: compression modulus (Figure 6), compressive strength (Figure 7) and compressive strain (Figure 8). Results given in the plots show the mean value obtained from 5 samples per each tested variant. Prior to the analysis, several results were rejected because they significantly differed from others. 

The plot in Figure 6 shows the mean values of the compression modulus of the samples seasoned in the six tested water environments. The highest mean compression modulus amounting to 57.20 MPa can be observed for the samples seasoned for 1 week in W3, i.e., in tap water containing 20 g/L of FeSO_4_. The lowest mean compression modulus was achieved for the samples seasoned for 3 months in W4, i.e., tap water containing 30 g/L of FeSO_4_. This value is 10.81 MPa.

Based on the plot (Figure 6), it can be concluded that the compression modulus decreases with an increase in the seasoning time. The difference between the highest and the lowest value of the compression modulus, depending on the water environment, is as follows:

W1—tap water—28.7%;
W2—tap water containing FeSO_4_ (10 g/L)—41.7%;
W3—tap water containing FeSO_4_ (20 g/L)—65.4%;
W4—tap water containing FeSO_4_ (30 g/L)—80.0%;
W5—tap water containing FeSO_4_ (50 g/L)—55.3%;
W6—“Zuber” mineral water—71.7%.

The highest difference can be observed for the samples seasoned in W4, whereas the lowest was observed for the samples seasoned in additive-free tap water (W1).

Analyzing the plotted compressive strength results of the samples seasoned in six water environments (Figure 7), it can be observed that the samples seasoned for 3 months in W6, i.e., “Zuber” mineral water, have the highest mean compressive strength, which is equal to 77.56 MPa. The lowest mean compressive strength was achieved by the samples seasoned for 3 months in W3, i.e., tap water containing 20 g/L of FeSO_4_. This value amounts to 59.20 MPa. The plot reveals that the compressive strengths of the samples seasoned in the tested water environments are similar. Nevertheless, certain differences can be observed. The biggest difference between the results can be observed for the samples seasoned in W3, and it amounts to 19.3%. The smallest difference between the results can be observed for the samples seasoned in W2, i.e., tap water containing 10 g/L of FeSO_4_, amounting to 0.5%. As for other samples seasoned in the tested water environments, the difference between the maximum and the minimum values is as follows:

W1—tap water—1.9%;
W4—tap water with the addition of FeSO_4_ (30 g/L)—5.9%;
W5—tap water with the addition of FeSO_4_ (50 g/L)—2.0%;
W6—“Zuber” mineral water—9.5%.

It is evident from the plot in Figure 7 that the compressive strength of the samples increases with an increase in the seasoning time in a given water environment. The compressive strength of the adhesive composition seasoned in tap water (W1) is higher than the compressive strength of the samples that were seasoned in water solutions containing iron (II) sulfate—FeSO_4_.

Analyzing the plot in Figure 8, it can be observed that the samples seasoned in W3 for 3 months and those seasoned in W1 for 1 month have the same highest mean compressive strain amounting to 8%. The lowest mean compressive strain of 6.42% was obtained for the samples seasoned for 1 week in W5—tap water containing 50 g/L of FeSO_4_. The differences between the compressive strains obtained for the samples seasoned in different water environments are as follows:

W1—tap water—0.5%;
W2—tap water with the addition of FeSO_4_ (10 g/L)—1.1%;
W3—tap water with the addition of FeSO_4_ (20 g/L)—1.2%;
W4—tap water with the addition of FeSO_4_ (30 g/L)—0.4%;
W5—tap water with the addition of FeSO_4_ (50 g/L—1.4%;
W6—“Zuber” mineral water—0.2%.

The highest difference can be observed for the samples seasoned in W5, i.e., the water environment with the highest content of iron (II) sulfate. This difference amounts to 1.4%. In contrast, the smallest difference between the compressive strain results can be observed for the samples seasoned in W6, i.e., mineral water. The plot demonstrates that the compressive strain of the tested samples increases with an increase in the seasoning time. 

In the conducted tests, both the type of water environment, including the concentration of iron sulfate, and the time of exposure were analyzed. Many publications analyze and influence the type of environment and the exposure time of both adhesives and adhesive joints. 

Many authors emphasize that moisture is the substance that causes the greatest difficulties in terms of environmental stability such as, for example, adhesives and other adhesive materials [15,17,21]. Seasoning in the water environment can be a serious problem, affecting the degradation of adhesives and adhesive joints, because this is due to the properties of water, which is a polar liquid that can permeate most polymers. In this way, it weakens the adhesive materials. 

Zanni-Deffarges and Shanahan [20] stated that the diffusion of water into the adhesive decreases its stiffness and mechanical resistance. In the carried out tests, it can be noted that the compression modulus decreased with an increase in the seasoning time. Sugiman et al. [15] also noted that the mechanical properties degraded in a linear way with the moisture content (exposure time). The tensile strength and elastic modulus decreased with increasing moisture content, while the strain tended to increase. Moreover the tensile strength is degraded more than the elastic modulus. Lai et al. [21] reported the hygro-mechanical response of a DGBA based epoxy as a function of moisture uptake. They noticed that water concentration affects both the diffusion kinetics and also epoxy resin properties. De Neve and Shanahan [17] stated that long-term exposure of the epoxy adhesive to water leaded to both reversible (physical) and irreversible (chemical) degradation of the material.

Lettieri and Frigione [24] stated that the amounts of absorbed water depended on the humidity level of exposure: the higher the relative humidity, the higher the water uptake; however, larger increases in absorbed water were noticed above 75% RH. The water ingress led to the plasticization of the adhesive, the enhanced the reactivation of cross-linking reactions and the erasure of physical ageing.

Frigione et al. [38] investigated the bond strength of epoxy adhesives and their efficiency when joining to concrete elements. Flexural tests were undertaken to determine the mechanical properties of the exposed and the control specimens of three different epoxy adhesives. Besides, the water resistance of concrete/concrete epoxy joints was investigated by comparing bond strength with those of the control samples; the maximum period of immersion was one month. The authors observed that the effect of water on the adhesion of the joints was found to be significant, especially at longer immersion times; the bond strength of concrete–adhesive specimens reduced by 30% after one month of immersion in water.

## 4. Conclusions 

Epoxy adhesive compounds are more and more often studied, which leads to the significant improvement of adhesive-bonded joints fabricated with the use of these compounds. This study investigated the effect of two operating factors: water and iron (II) sulfate—FeSO_4_ on the mechanical properties of an adhesive compound made of Epidian 53 epoxy resin combined with triethylenetetramine curing agent (Z-1 trade name) in a proper stoichiometric ratio.

Taking into account obtained strength test results with respect to seasoning time and water environment, the following conclusions can be drawn:

- The mean compression modulus of individual samples decreases with an increase in the seasoning time;
- The mean compressive strength of individual samples increases with an increase in the seasoning time;
- The mean compressive strain of individual samples increases with an increase in the seasoning time;
- The samples seasoned in the tested water environments for 1 week have higher compression modulus than those seasoned for a longer period of time. The difference between the minimum and maximum compression modulus is very big. With an increase of the iron content in water, the increase in the compression modulus is the highest during the first week of seasoning;
- It has been observed that a lower iron content in water has a more significant impact on increasing the adhesive compound compressive strength than a higher iron content or lack thereof;
- It has been found that the samples seasoned in water with lower iron contents have lower compressive strains, whereas high iron contents lead to increased compressive strains.

The results point to increased strength properties of the samples that were seasoned in water with a low iron content. The study has found that if the iron content in water is too high, it has a negative effect on the mechanical properties of the tested adhesive compounds. To ensure the highest mechanical properties possible, adhesive-bonded joints fabricated with Epidian 53 epoxy resin and triethylenetetramine curing agent must be seasoned in tap water or water with low iron content. More detailed studies must be performed in order to determine the most suitable iron content.

## Figures and Tables

**Table 1:** Properties of Epidian 53 epoxy resin before curing [34].

Properties	Value
Viscosity at 20 °C [mPa·s]	900–1500
Density at 20 °C [g/cm^3^]	1.11–1.15
Gel time (10 g of Epidian 53 epoxy resin and 1.05 g of Z-1 curing agent) at 20 °C [min]	200
Thermal spike at 50 °C	130–170
Substances insoluble in acetone [%] not less than	0.03
Epoxy number [mol/100 g]	0.41
Boiling point [°C]	1410
Flash point [°C]	75

**Table 2:** Properties of Epidian 53 epoxy resin after curing [34].

Properties	Value
Peel strength [MPa]	24.52
Peel stresses [MPa]	40–60
Bending strength [MPa]	80–100
Compressive strength [MPa]	70–90
Shear strength of adhesive layer cured for 16 h at 20–25 °C, 6h at 80 ± 2 °C [MPa], not less than	7.84
Shear strength of adhesive layer cured for 7 days at 20–25 °C [MPa], not less than	5.86

**Table 3:** Properties of triethylenetetramine curing agent [34].

Properties	Value
Viscosity at 25 °C [mPa·s]	20–30
Density at 20 °C [g/cm^3^]	0.978–0.983
Amine number [mgKOH/g]	min. 1100
Boiling point at 1013 hPa	277.5 °C
Boiling point at 66.5 hPa	183 °C
Boiling point at 13 hPa	143 °C

**Table 4:** Water environments.

No.	Denotation	Water	Additive	Content [g/L]
**1**	W1	Tap water	-	0
**2**	W2	Tap water	FeSO_4_	10
**3**	W3	Tap water	FeSO_4_	20
**4**	W4	Tap water	FeSO_4_	30
**5**	W5	Tap water	FeSO_4_	50
**6**	W6	Mineral water “Zuber”	-	-

**Table 5:** Selected physical and chemical parameters of tap water, 1st quarter 2018 (based on [35]).

Parameter	Unit	Content/Value
Chlorides	mg/L	29.4
Fluorides	mg/L	<0.4
Magnesium	mg/L	23.0
Sulfates	mg/L	36.4
Sodium	mg/L	9.2
Iron	μg/L	46
Calcium	mg/L	98
Hardness	mval/L	381

**Table 6:** Physical and chemical properties of iron (II) sulfate (based on [36]).

Properties	Description/Value
Chemical formula	FeSO_4_
Physical state	solid (crystal)
Color	green-blue
Odor	odorless
pH-value in 50 g/L	from 3 to 4
Melting/freezing point	>60 °C
Flammability	non-flammable
Density at 20 °C	1.89 g/cm^3^
Breakdown temperature	>300 °C

**Table 7:** Minerals in “Zuber” mineral water (based on [37]).

Cations [mg/L]		Anions [mg/L]	
Sodium	5821.00	Bicarbonates	16,593.00
Calcium	85.52	Free carbon dioxide	1700.00
Magnesium	341.50	Chlorides	396.90
Potassium	252.60	Bromides	0.30
Iron	0.87	Iodides	0.04
Lithium	43.67	Sulfates	5.30
		Metasilicic acid	35.23
		Metaboric acid	7.61

**Table 8:** The pH values of the water solutions used in experiments.

Water Denotation	pH Value	Reaction
W1	7	Neutral
W2	4	Acidic
W3	4	Acidic
W4	3	Strongly acidic
W5	3	Strongly acidic
W6	8	Basic
